# Supplementary material for: Cost-Effectiveness Analysis of an Intracranial Stereotactic Radiotherapy Service for Brain Metastasis in a North Queensland Regional Cancer Centre
Source: Cancers (Basel). 2026 Jan 2;18(1):163. doi: 10.3390/cancers18010163 (PMC12785005; doi:10.3390/cancers18010163)
Supplement: Supplementary file 1 [file cancers-18-00163-s001.zip › cancers-4044072-supplementary.pdf]

Supplementary Materials File S1

Supplementary Materials File S1: Cost Sources

Flight round trip cost in 2025 to Brisbane				
	Average	Min	Max	https://www.kayak.com.au/flight-routes/Townsville-TSV/Brisbane-BNE, accessed on 03 July 2025
Townsville	333	268	398	https://www.kayak.com.au/flight-routes/Cairns-CNS/Brisbane-BNE, accessed on 03 July 2025
Cairns	246	214	278	https://www.kayak.com.au/flight-routes/Mackay-MKY/Brisbane-BNE, accessed on 03 July 2025
Mackay	268	215	321	https://www.kayak.com.au/flight-routes/Mount-Isa-ISA/Brisbane-BNE, accessed on 03 July 2025
Mount Isa	848.5	498	1199	https://www.expedia.com.au/lp/flights/cnj/bne/cloncurry-to-brisbane, accessed on 03 July 2025
Cloncurry	1047	963	1131	https://www.kayak.com.au/flight-routes/Townsville-TSV/Brisbane-BNE, accessed on 03 July 2025
Flight round trip cost in 2025 to Townsville				
	Average	Min	Max	https://www.kayak.com.au/flight-routes/Cairns-CNS/Townsville-TSV, accessed on 03 July 2025
Cairns	501	389	612	https://www.kayak.com.au/flight-routes/Mackay-MKY/Townsville-TSV, accessed on 03 July 2025
Mackay	506	402	610	https://www.kayak.com.au/flight-routes/Mount-Isa-ISA/Townsville-TSV, accessed on 03 July 2025
Mount Isa	809	381	1237	https://www.skyscanner.com.au/routes/cnj/tsv/cloncurry-to-townsville.html#:~:text=How%20to%20find%20the%20cheapest,flights%20from%20CNJ%20to%20TSV. , accessed on 03 July 2025
Cloncurry	534			https://www.skyscanner.com.au/routes/wtb/tsv/brisbane-west-wellcamp-to-townsville.html, accessed on 03 July 2025
Toowoomba Wellcamp	966	762	1169	https://www.kayak.com.au/flight-routes/Cairns-CNS/Townsville-TSV, accessed on 03 July 2025

Caregiver cost (All employees average weekly total earnings-seasonally adjusted)				
		Weekly earnings in May	Daily	
	2021	1305.8	187	https://www.abs.gov.au/statistics/labour/earnings-and-working-conditions/average-weekly-earnings-australia/nov-2024, accessed on 03 July 2025
	2022	1344.7	192	https://www.abs.gov.au/statistics/labour/earnings-and-working-conditions/average-weekly-earnings-australia/nov-2024, accessed on 03 July 2025
	2023	1399.1	200	https://www.abs.gov.au/statistics/labour/earnings-and-working-conditions/average-weekly-earnings-australia/nov-2024, accessed on 03 July 2025
	2024	1510.9	216	https://www.abs.gov.au/statistics/labour/earnings-and-working-conditions/average-weekly-earnings-australia/nov-2024, accessed on 03 July 2025
Accommodation				
Brisbane	299			https://www.kayak.com.au/Brisbane-Hotels.27249.hotel.ksp, accessed on 03 July 2025
Townsville	202			https://www.kayak.com.au/Townsville-Hotels.11286.hotel.ksp, accessed on 03 July 2025
Taxi				
Taxi	Min cost weekdays			Passenger fares (taxis)—South East Queensland, accessed on 03 July 2025

	3.65 + 2.62 per km			
Ferry	18			https://magneticislandferries.com.au/prices-fares/, accessed on 03 July 2025
Consumer price index				
Year	Travel	Overall	Health	
2025	129.9	170.8	170.8	https://www.abs.gov.au/statistics/economy/price-indexes-and-inflation/consumer-price-index-australia/latest-release#selected-tables-capital-cities, accessed on 03 July 2025
2024	131.2	137.4	164.1	https://www.abs.gov.au/statistics/economy/price-indexes-and-inflation/consumer-price-index-australia/latest-release#selected-tables-capital-cities, accessed on 03 July 2025
2023	126.7	132.6	137.6	https://www.abs.gov.au/statistics/economy/price-indexes-and-inflation/consumer-price-index-australia/latest-release#selected-tables-capital-cities, accessed on 03 July 2025
